# Grey Situation Group Decision-Making Method Based on Prospect Theory

**DOI:** 10.1155/2014/703597

**Published:** 2014-08-12

**Authors:** Na Zhang, Zhigeng Fang, Xiaqing Liu

**Affiliations:** ^1^Corps Financial Development Research Center, Shihezi University, Wujiaqu 831300, China; ^2^Commerce College, Shihezi University, Wujiaqu 831300, China; ^3^College of Economics and Management, Nanjing University of Aeronautics and Astronautics, Nanjing 210016, China

## Abstract

This paper puts forward a grey situation group decision-making method on the basis of prospect theory, in view of the grey situation group decision-making problems that decisions are often made by multiple decision experts and those experts have risk preferences. The method takes the positive and negative ideal situation distance as reference points, defines positive and negative prospect value function, and introduces decision experts' risk preference into grey situation decision-making to make the final decision be more in line with decision experts' psychological behavior. Based on TOPSIS method, this paper determines the weight of each decision expert, sets up comprehensive prospect value matrix for decision experts' evaluation, and finally determines the optimal situation. At last, this paper verifies the effectiveness and feasibility of the method by means of a specific example.

## 1. Introduction

Grey situation decision is a method to choose the decision with optimal effect from multiple decisions and objectives, with the prerequisite that decision information should have grey elements [1–3]. Since Professor Dengjulong [1] put forward grey decision-making theory, numerous experts and scholars at home and abroad conduct researches and applications on the theory.

In the aspect of improvement for grey situation model, [4] applies grey situation decision-making to the situation of decision information as interval number on the basis of analysis on distance of interval number and grey relation entropy and proposes optimization methods for objective weight of grey situation decision-making. Reference [5] defines parameter setting for intelligence algorithm as a uniform design problem and makes decisions for uniform designed parameter combination by means of grey situation decision-making. Reference [6] brings up a multiobjective grey situation decision-making method based on prospect theory, in consideration of the influences of decision experts' risk attitude to multiobjective decision. Reference [7] comes up with a linear transformation operator with bonus and forfeit property and gives multiobjective interval number grey situation decision-making method. Reference [8] explores the case of assessment information as interval grey number and sets up an objective weight optimization model for multiobjective grey situation decision-making. Reference [9] proposes a weighting method for objective weight of grey situation decision-making, in response to the question that equal weight method for grey situation decision-making is unable to reflect decision experts' preference and practical situation of decision-making process. Reference [10] analyzes the generality of grey situation decision-making using general system theory, defines grey general situation decision-making, and develops its mathematical model.

In recent years, the grey situation model has made a lot of achievements in application field too. Reference [11] applies grey situation decision-making method to stock market. Reference [12] conducts performance assessment for medical industry by means of grey situation decision-making method. Reference [13] assumes that all the attribute indexes are in the state of trapezoid fuzzy numbers and makes optimization for site selection alternatives by grey situation decision-making method. Reference [14] develops optimal model for artillery fire scheme on the basis of grey situation decision-making method, aiming at solving the problems of information uncertainty and information incompleteness in the battlefield. Reference [15] suggests a general maintenance resource integration method based on grey situation decision-making theory, on account of growing number of maintenance resource categories for troops equipment and increasingly heavy security burden. Reference [16] evaluates supply chain partnership using 9 d cobweb model and solves a variety of comprehensive evaluations with different polarity objectives by means of grey decision-making theory.

Throughout relevant researches on grey situation decision-making, it is found that the situation that multiple decision experts participate in decision-making and those decision experts have risk preference is rarely considered. However, in practical decision-making process, especially in the case that the scheme set has numerous qualitative indexes, multiple decision experts should participate in decision-making and inevitably their subjective preference will influence the final decision results. Therefore, this paper makes use of prospect theory [17, 18] to deal with decision expert sample matrix and reveals the decision experts' decision psychology and risk attitudes that contribute to risk aversion in choices involving sure gains and risk seeking in choices involving sure losses. Meanwhile, as for the question that it is hard to determine experts' weight in group decision, this paper develops the optimal and the worst prospect value matrix for decision experts situation, gets decision experts' weight based on TOPSIS method, collects experts opinions, sorts situations of each event, and finally gets the optimal situation. This model provides a feasible and practical method for grey situation group decision-making in consideration of decision experts' risk preference.

## 2. Main Methods and Results

### 2.1. Problem Description

In a grey situation group decision-making problem, the event set is denoted as *A* = {*a*_1_, *a*_2_,…, *a*_*n*_}, the strategy set as *B* = {*b*_1_, *b*_2_,…, *b*_*m*_}, the decision-making experts as *D* = {*d*_1_, *d*_2_,…, *d*_*p*_}, the *k*th decision expert's situation set as *S* = {*s*_*ij*_^*k*^ = (*a*_*i*_^*k*^, *b*_*j*_^*k*^)∣*a*_*i*_^*k*^ ∈ *A*, *b*_*j*_^*k*^ ∈ *B*}, and the effect sample value of the situation *s*_*ij*_^*k*^ ∈ *S* given by the *k*th decision expert under the target *l* as *μ*_*ij*_^*k*(*l*)^(⊗)(*i* = 1,2,…, *n*; *j* = 1,2,…, *m*; *l* = 1,2,…, *s*; *k* = 1,2,…, *p*), then there are the objective weight vector of *s*_*ij*_^*k*^ for the* k*th decision expert's situation as *W* ^*k*^ = {*ω*_1_^*k*^, *ω*_2_^*k*^,…, *ω*_*s*_^*k*^} which satisfies ∑_*l*=1_^*s*^ *ω*_*l*_^*k*^ = 1, and the weight vector of decision experts as *W* ^*k*^ = {*ω*_1_^*k*^, *ω*_2_^*k*^,…, *ω*_*s*_^*k*^} which satisfies ∑_*k*=1_^*p*^ *ω*_*k*_ = 1. This paper mainly discusses how to determine the optimal situation for grey decision-making in the situation that multiple decision experts participate in decision-making process.

The effect sample value matrix of the *k*th decision expert situation *s*_*ij*_^*k*^ under the objective *l* can be expressed in the following way:

(1) $$\begin{array}{c} U_{1}^{k(l)}\left(⊗\right)=\left[\begin{array}{cccc} \left[{\underline{\mu }}_{11}^{k(l)},{\overset{-}{\mu }}_{11}^{k(l)}\right] & \left[{\underline{\mu }}_{12}^{k(l)},{\overset{-}{\mu }}_{12}^{k(l)}\right] & \cdot \cdot \cdot & \left[{\underline{\mu }}_{1m}^{k(l)},{\overset{-}{\mu }}_{1m}^{k(l)}\right]\\ \left[{\underline{\mu }}_{21}^{k(l)},{\overset{-}{\mu }}_{21}^{k(l)}\right] & \left[{\underline{\mu }}_{22}^{k(l)},{\overset{-}{\mu }}_{22}^{k(l)}\right] & \cdot \cdot \cdot & \left[{\underline{\mu }}_{2m}^{k(l)},{\overset{-}{\mu }}_{2m}^{k(l)}\right]\\ \cdot \cdot \cdot & \cdot \cdot \cdot & \cdot \cdot \cdot & \cdot \cdot \cdot \\ \left[{\underline{\mu }}_{n1}^{k(l)},{\overset{-}{\mu }}_{n1}^{k(l)}\right] & \left[{\underline{\mu }}_{n2}^{k(l)},{\overset{-}{\mu }}_{n2}^{k(l)}\right] & \cdot \cdot \cdot & \left[{\underline{\mu }}_{nm}^{k(l)},{\overset{-}{\mu }}_{nm}^{k(l)}\right] \end{array}\right]. \end{array}$$

**Definition 1 .**

Given the corresponding value of $\mu_{ij}^{+k(l)}=\max\;\max\left\{({\underline{\mu }}_{ij}^{k(l)}+{\overset{-}{\mu }}_{ij}^{k(l)})/2\right\}$(1 ≤ *i* ≤ *n*; 1 ≤ *j* ≤ *m*; *l* = 1,2,…, *s*; *k* = 1,2,…, *p*) as $\left[{\underline{\mu }}_{ij}^{+k(l)},{\overset{-}{\mu }}_{ij}^{+k(l)}\right]$, *μ*_*ij*_^+*k*(*l*)^ is the positive ideal value measure of the *k*th decision expert situation *s*_*ij*_^*k*^ under the objective *l*.

Given the corresponding value of $\mu_{ij}^{-k(l)}=\min\;\min\left\{({\underline{\mu }}_{ij}^{k(l)}+{\overset{-}{\mu }}_{ij}^{k(l)})/2\right\}\;\;(k=1,2,\ldots ,p)$ as $\left[{\underline{\mu }}_{ij}^{-k(l)},{\overset{-}{\mu }}_{ij}^{-k(l)}\right]$, *μ*_*ij*_^−*k*(*l*)^ is the negative ideal value measure of the *k*th decision expert situation *s*_*ij*_^*k*^ under the objective *l*.

**Definition 2 .**

Given the value measure of the *k*th decision expert situation *s*_*ij*_^*k*^ under the objective *l* as *μ*_*ij*_^*k*(*l*)^, the positive ideal value measure as *μ*_*ij*_^+*k*(*l*)^, and the negative ideal value measure as *μ*_*ij*_^−*k*(*l*)^, then (2) and (3) are positive and negative ideal situation distance of the *k*th decision expert situation *s*_*ij*_^*k*^ under the objective *l*, respectively. Consider

(2) $$\begin{array}{c} d\left(\mu_{ij}^{k(l)},\mu_{ij}^{+k(l)}\right)=\sqrt{{{\left[{\underline{\mu }}_{ij}^{+k(l)}-{\overset{-}{\mu }}_{ij}^{k(l)}\right]}^{2}+[{\overset{-}{\mu }}_{ij}^{+k(l)}-{\underline{\mu }}_{ij}^{k(l)}]}^{2}}, \end{array}$$

(3) $$\begin{array}{c} d\left(\mu_{ij}^{k(l)},\mu_{ij}^{-k(l)}\right)=\sqrt{{{\left[{\underline{\mu }}_{ij}^{-k(l)}-{\overset{-}{\mu }}_{ij}^{k(l)}\right]}^{2}+[{\overset{-}{\mu }}_{ij}^{k(l)}-{\underline{\mu }}_{ij}^{k(l)}]}^{2}}. \end{array}$$

### 2.2. Prospect Value of Grey Situation Effect Measure

#### 2.2.1. Efficiency Index

Assuming that the effect measure of the situation *s*_*ij*_^*k*^ given by the *k*th decision expert is efficiency index, if negative ideal situation distance is taken as reference point, the situation distance of alternatives is superior to that of negative ideal scheme and, at this time, psychological perception of the decision expert is gains. The value function is *v* ^+^[*s*_*ij*_^−*k*(*l*)^] = [*d*(*μ*_*ij*_^*k*(*l*)^, *μ*_*ij*_^−*k*(*l*)^)]^*α*^.

However, the situation distance of alternatives cannot be worse than that of negative ideal scheme. That is to say, if the situation distance of negative ideal is taken as reference point, its value function will become *v* ^−^[*s*_*ij*_^−*k*(*l*)^] = 0.

If the situation distance of positive ideal is taken as reference point, the situation distance of alternatives will be worse than that of positive ideal scheme. At this time, psychological perception of decision expert is risks. The value function is *v* ^−^[*s*_*ij*_^+*k*(*l*)^] = −*θ*[−*d*(*μ*_*ij*_^*k*(*l*)^, *μ*_*ij*_^+*k*(*l*)^)]^*β*^.

However, the situation distance of alternatives cannot be superior to that of positive ideal scheme. That is to say, if the situation distance of positive ideal is taken as reference point, its value function will become *v* ^+^[*s*_*ij*_^+*k*(*l*)^] = 0.

#### 2.2.2. Cost Index

Assuming that the effect measure of the situation *s*_*ij*_^*k*^ given by the *k*th decision expert is cost index, if positive ideal situation distance is taken as reference point, the situation distance of alternatives is superior to that of positive ideal scheme and, at this time, psychological perception of decision expert is gains. The value function is *v* ^+^[*s*_*ij*_^+*k*(*l*)^] = [*d*(*μ*_*ij*_^*k*(*l*)^, *μ*_*ij*_^+*k*(*l*)^)]^*α*^.

However, the situation distance of alternatives cannot be superior to that of positive ideal scheme. That is to say, if the situation distance of positive ideal is taken as reference point, its value function will become *v* ^−^[*s*_*ij*_^+*k*(*l*)^] = 0.

If the situation distance of negative ideal is taken as reference point, the situation distance of alternatives will be worse than that of positive ideal scheme. At this time, psychological perception of decision expert is risks. The value function is *v* ^−^[*s*_*ij*_^−*k*(*l*)^] = −*θ*[−*d*(*μ*_*ij*_^*k*(*l*)^, *μ*_*ij*_^−*k*(*l*)^)]^*β*^.

However, the situation distance of alternatives cannot be worse than that of negative ideal scheme. That is to say, if the situation distance of negative ideal is taken as reference point, its value function will become *v* ^+^[*s*_*ij*_^−*k*(*l*)^] = 0.

No matter in the case of efficiency index or cost index, both *v* ^+^[*s*_*ij*_^+*k*(*l*)^] and *v* ^+^[*s*_*ij*_^−*k*(*l*)^] show that the psychological perception of decision expert for alternatives measure is risk aversion and is expressed as positive value of gains. Both *v* ^−^[*s*_*ij*_^+*k*(*l*)^] and *v* ^−^[*s*_*ij*_^−*k*(*l*)^] reveal that the psychological perception of decision expert for alternatives measure is risk seeking and is expressed as negative value of losses. Parameters *α* and *β* express the concave and convex degree of gains and losses value power function, respectively, and *α*, *β* < 1 shows decision experts' sensitivity is decreasing. *θ* expresses that the losses curve is much deeper than the gains curve, and *θ* > 1 reveals loss aversion [17].

No matter in the case of efficiency index or cost index, the prospect value by taking positive and negative ideal situation distance as different reference points is positive value of gains and negative value of risks in decision experts' psychological perception. In order to eliminate the influences of dimension to calculation results, the prospect value gotten from positive and negative ideal situation as different reference points is normalized as follows:

(4) $$\begin{array}{c} {\overset{-}{v}}^{\pm }\left[s_{ij}^{\pm k(l)}\right]=\frac{v^{\pm }\left[s_{ij}^{\pm k(l)}\right]}{\max_{i=1,2,\ldots ,n}\left|v^{\pm }\left[s_{ij}^{\pm k(l)}\right]\right|}. \end{array}$$

Normalized prospect value ${\overset{-}{v}}^{\pm }\left[s_{ij}^{\pm k(l)}\right]\;\;(i=1,2,\ldots ,n$; *j* = 1,2,…, *m*; *l* = 1,2,…, *s*; *k* = 1,2,…, *p*) has the following properties: (1) ${\overset{-}{v}}^{\pm }[s_{ij}^{\pm k(l)}]$ is dimensionless; (2) the better the situation, the bigger ${\overset{-}{v}}^{\pm }[s_{ij}^{\pm k(l)}]$, and the worse the situation, the smaller the ${\overset{-}{v}}^{\pm }[s_{ij}^{\pm k(l)}]$; (3) ${\overset{-}{v}}^{+}[s_{ij}^{\pm k(l)}]\in [0,1]$, ${\overset{-}{v}}^{-}[s_{ij}^{\pm k(l)}]\in [-1,0]$.

**Proof**

Consider the following

*Case* (*i*). For ${\overset{-}{v}}^{+}[s_{ij}^{\pm k(l)}]=v^{+}[s_{ij}^{\pm k(l)}]/\max_{i=1,2,\ldots ,n}v^{+}[s_{ij}^{\pm k(l)}]$, considering that 0 ≤ *v* ^+^[*s*_*ij*_^±*k*(*l*)^] ≤ max⁡_*i*=1,2,…,*n*_ *v* ^+^[*s*_*ij*_^±*k*(*l*)^], the better the *v* ^+^[*s*_*ij*_^±*k*(*l*)^], the bigger the ${\overset{-}{v}}^{+}[s_{ij}^{\pm k(l)}]$, and ${\overset{-}{v}}^{+}[s_{ij}^{\pm k(l)}]\in [0,1]$. Thus, properties (1), (2), and (3) are proved.

*Case* (*ii*). For ${\overset{-}{v}}^{-}[s_{ij}^{\pm k(l)}]=-v^{-}[s_{ij}^{\pm k(l)}]/\min_{i=1,2,\ldots ,n}v^{-}[s_{ij}^{\pm k(l)}]$, considering that −min⁡_*i*=1,2,…,*n*_ *v* ^−^[*s*_*ij*_^±*k*(*l*)^] ≤ *v* ^−^[*s*_*ij*_^±*k*(*l*)^] ≤ 0, the worse the *v* ^−^[*s*_*ij*_^±*k*(*l*)^], the smaller the ${\overset{-}{v}}^{\pm }[s_{ij}^{\pm k(l)}]$, and ${\overset{-}{v}}^{-}[s_{ij}^{\pm k(l)}]\in [-1,0]$. Thus, properties (1), (2), and (3) are proved.

According to the value function given by [17], assuming that the prospect weight that decision experts have when facing gains and losses is *π* ^+^(*ω*_*l*_^*k*^) and *π* ^−^(*ω*_*l*_^*k*^), respectively, the comprehensive prospect value of the scheme is the sum of positive and negative prospect values. Suppose that decision experts take the positive and negative ideal situation distance of the situation *s*_*ij*_^*k*^ under objective *l* as different references points and meanwhile they think the distance between reference points and the positive ideal situation is as important as that between reference points and the negative ideal situation distance, and then comprehensive prospect value based on positive and negative ideal situation distance as two reference points can be defined as follows:

(5) Vijk={∑l=1sv−+[sij−k(l)]·π+(ωlk)+∑l=1sv−−[sij−k(l)]·π−(ωlk)} +{∑l=1sv−+[sij+k(l)]·π+(ωlk)+∑l=1sv−−[sij+k(l)]·π−(ωlk)}=∑l=1sv−+[sij−k(l)]·π+(ωlk)+∑l=1sv−−[sij+k(l)]·π−(ωlk),π+(ωlk)=(ωlk)γ{(ωlk)γ+[1−(ωlk)]γ}1/γ,π−(ωlk)=(ωlk)δ{(ωlk)δ+[1−(ωlk)]δ}1/δ.

Therefore, the comprehensive prospect based on positive and negative ideal situation distance as two reference points is the sum of positive prospect value taking negative ideal situation distance as reference point and negative prospect value taking positive ideal situation distance as reference point.

According to [17], the parameters of prospect value function and weight function in this paper are consistent with practical empirical data as *α* = *β* = 0.88, *θ* = 2.25, *γ* = 0.61, and *δ* = 0.69.

### 2.3. Determination of Decision Experts' Weight Vector

**Definition 3 .**

Assume that *V* ^+^ = {*V*_1*j*_^+^, *V*_2*j*_^+^,…, *V*_*nj*_^+^} is the optimal prospect value matrix of all the decision experts' decision, namely, the optimal situation matrix of the situations for all decision experts' choices, where *V*_*ij*_^+^ = max⁡_*k*=1,2,…,*p*_ *V*_*ij*_^*k*^  (*i* = 1,2,…, *n*; *j* = 1,2,…, *m*).

Assume that *V* ^−^ = {*V*_1*j*_^−^, *V*_2*j*_^−^,…, *V*_*nj*_^−^} is the worst prospect value matrix of all the decision experts' decision, namely, the worst situation matrix of the situations for all decision experts' choices, where *V*_*ij*_^−^ = min⁡_*k*=1,2,…,*p*_ *V*_*ij*_^*k*^  (*i* = 1,2,…, *n*; *j* = 1,2,…, *m*).

**Definition 4 .**

Given *V* ^*k*^ = {*V*_1*j*_^*k*^, *V*_2*j*_^*k*^,…, *V*_*nj*_^*k*^} is the prospect value matrix of the *k*th decision expert's situation *s*_*ij*_^*k*^, then

(6) d(Vk,V+)=∑j=1m(V1jk−V1j+)2+∑j=1m(V2jk−V2j+)2+⋯+∑j=1m(Vnjk−Vnj+)2

is the distance between the prospect value matrix of the *k*th decision experts' situation and the optimal prospect value matrix. Given *V* ^*k*^ = {*V*_1*j*_^*k*^, *V*_2*j*_^*k*^,…, *V*_*nj*_^*k*^} is the prospect value matrix of the *k*th decision expert situation *s*_*ij*_^*k*^, then

(7) d(Vk,V−)=∑j=1m(V1jk−V1j−)2+∑j=1m(V2jk−V2j−)2+⋯+∑j=1m(Vnjk−Vnj−)2

is the distance between the prospect value matrix of the *k*th decision experts' situation and the worst prospect value matrix.

In order to avoid one-sidedness of evaluation results, make full use of decision information and keep the consistency of decision results; the greatest weight is given to the decision expert whose matrix is closest with the optimal prospect value and is the most far away from the worst prospect value matrix, and vice versa. This paper takes the optimal prospect value matrix as the optimal situation and the worst prospect value matrix as the worst situation and calculates the prospect value close degree of decision experts' situation according to traditional TOPSIS concept, and in view of the distance between prospect value matrix of multiple decision experts and the optimal and the worst prospect value matrix. Consider

(8) $$\begin{array}{c} C_{k}^{*}=\frac{d\left(V_{}^{k},V_{}^{+}\right)}{d\left(V_{}^{k},V_{}^{+}\right)+d\left(V_{}^{k},V_{}^{-}\right)}. \end{array}$$

Then, the weight of the *k*th decision expert is

(9) $$\begin{array}{c} \lambda_{k}=\frac{C_{k}^{*}}{\sum_{k=1}^{p}C_{k}^{*}}. \end{array}$$

And then, the comprehensive prospect value matrix based on the weight vector of decision experts is

(10) $$\begin{array}{c} V=\left[v_{ij}\right]=\overset{p}{\underset{k=1}{\sum }}\lambda^{p}\cdot V_{ij}^{k}. \end{array}$$

**Definition 5 .**

Given the comprehensive prospect value matrix *V*, if max⁡_1≤*j*≤*m*_{*v*_*ij*_} = *v*_*ij*_0__, *b*_*j*_0__ is optimal strategy for the event *a*_*i*_; if max⁡_1≤*j*≤*m*_{*v*_*ij*_} = *v*_*i*_0_*j*_, *a*_*i*_0__ is the corresponding optimal event of strategy *b*_*j*_; if max⁡_1≤*j*≤*m*_{*v*_*ij*_} = *v*_*i*_0_*j*_0__, *s*_*i*_0_*j*_0__ is the optimal situation.

### 2.4. Decision-Making Procedure

To sum up, the procedure of grey situation group decision-making method based on prospect theory is as follows.

**Step 1 .**

According to the event set *A* = {*a*_1_, *a*_2_,…, *a*_*n*_}, the strategy set *B* = {*b*_1_, *b*_2_,…, *b*_*m*_}, and decision experts set *D* = {*d*_1_, *d*_2_,…, *d*_*p*_}, the situation set of multiple decision experts is *S* = {*s*_*ij*_^*k*^ = (*a*_*i*_^*k*^, *b*_*j*_^*k*^)∣*a*_*i*_^*k*^ ∈ *A*, *b*_*j*_^*k*^ ∈ *B*}.

**Step 2 .**

Determine the decision objective and give corresponding effect sample matrix of multiple decision experts for objective *l* = 1,2,…, *s*.

**Step 3 .**

In light of Definitions 1 and 2, calculate the positive and negative ideal situation distance. Take positive and negative ideal situation distances as different reference points and calculate and normalize the positive and negative prospect value matrix.

**Step 4 .**

Give target weight vectors of the situation given by each decision expert, plug those target weight vectors into formula (5), and get comprehensive prospect matrix based on two reference points of positive and negative ideal situation distances.

**Step 5 .**

According to Definition 3, determine the optimal and worst prospect value matrix of all decision experts. And in light of Definition 4, calculate the distances between the prospect value matrix of multiple decision experts situation and the optimal and the worst prospect value matrix.

**Step 6 .**

According to formulas (8) and (9), calculate the weight of experts' decisions. In light of formula (10), get comprehensive prospect value matrix based on the matrix of decision experts' weight vector, and finally determine the optimal situation.

## 3. Example Analysis

A new round of aid program for Xinjiang is an important decision and strategic deployment made by the Party Central Committee and the State Council to promote Xinjiang's development and maintain Xinjiang's social stability, requiring that 19 provinces and cities in the mid-east region aid Xinjiang's development. Since March 2010, new industry production lines in Xinjiang are increasing day by day. Suppose that there are 3 production lines in an industry area, 4 suppliers are shortlisted in selecting suppliers, and there are 3 experts in decision-making expert group.

**Step 1 .**

Choose suppliers' choices in 3 production lines as the events, and the event set is *A* = {*a*_1_, *a*_2_, *a*_3_}. Supplier 1, supplier 2, supplier 3, and supplier 4 constitute the strategy set *B* = {*b*_1_, *b*_2_, *b*_3_, *b*_4_}. Expert 1, expert 2, and expert 3 constitute the decision expert set *D* = {*d*_1_, *d*_2_, *d*_3_}. The event set, strategy set, and decision expert set constitute decision experts' situation set *S* = {*s*_*ij*_^*k*^ = (*a*_*i*_^*k*^, *b*_*j*_^*k*^)∣*a*_*i*_^*k*^ ∈ *A*, *b*_*j*_^*k*^ ∈ *B*, *k* ∈ *D*; *i* = 1,2, 3, *j* = 1,2, 3,4, *k* = 1,2, 3}.

**Step 2 .**

Determine decision-making objective. After several rounds of specialist researches, take quality, design, and price as decision-making objectives, where quality and design are qualitative index. Decision experts make assessment by means of expert scoring method. The higher the score is, the more uncertain the index is. Decision experts give scores between 0 and 10. Price is cost index; the lower the data which the suppliers provided, the better the decision. Effect sample value matrix given by multiple experts with regard to the quality and design of decision-making objectives is as follows:

(11) $$\begin{array}{c} U_{1}^{1(1)}=\left[\begin{array}{cccc} \left[5,7\right] & \left[6,7\right] & \left[5,6\right] & \left[7,9\right]\\ \left[3,5\right] & \left[4,6\right] & \left[4,6\right] & \left[3,4\right]\\ \left[5,6\right] & \left[4,6\right] & \left[6,8\right] & \left[5,7\right] \end{array}\right],\\ U_{1}^{1(2)}=\left[\begin{array}{cccc} \left[4,5\right] & \left[5,7\right] & \left[4,6\right] & \left[3,5\right]\\ \left[5,6\right] & \left[4,5\right] & \left[4,7\right] & \left[4,6\right]\\ \left[5,7\right] & \left[3,6\right] & \left[7,9\right] & \left[4,5\right] \end{array}\right],\\ U_{1}^{2(1)}=\left[\begin{array}{cccc} \left[5,6\right] & \left[6,8\right] & \left[5,6\right] & \left[8,9\right]\\ \left[3,4\right] & \left[4,6\right] & \left[4,5\right] & \left[4,5\right]\\ \left[4,6\right] & \left[5,7\right] & \left[7,9\right] & \left[5,6\right] \end{array}\right],\\ U_{1}^{2(2)}=\left[\begin{array}{cccc} \left[4,6\right] & \left[6,7\right] & \left[4,5\right] & \left[3,6\right]\\ \left[5,7\right] & \left[4,6\right] & \left[4,7\right] & \left[4,7\right]\\ \left[5,8\right] & \left[4,6\right] & \left[8,10\right] & \left[5,8\right] \end{array}\right],\\ U_{1}^{3(1)}=\left[\begin{array}{cccc} \left[4,5\right] & \left[6,7\right] & \left[5,7\right] & \left[8,9\right]\\ \left[3,4\right] & \left[4,5\right] & \left[3,4\right] & \left[4,6\right]\\ \left[4,5\right] & \left[5,6\right] & \left[7,8\right] & \left[3,5\right] \end{array}\right],\\ U_{1}^{3(2)}=\left[\begin{array}{cccc} \left[3,4\right] & \left[5,6\right] & \left[4,6\right] & \left[3,4\right]\\ \left[5,6\right] & \left[4,6\right] & \left[5,7\right] & \left[4,5\right]\\ \left[7,8\right] & \left[4,7\right] & \left[8,9\right] & \left[6,8\right] \end{array}\right]. \end{array}$$

The decision objective target is an effect sample value matrix of suppliers' offers. Because this is not assessed by decision experts, the sample value matrix of target effect is the same. Consider

(12) $$\begin{array}{c} U_{1}^{1,2,3(3)}=\left[\begin{array}{cccc} 8 & 7 & 9 & 8\\ 15 & 12 & 13 & 14\\ 11 & 13 & 13 & 10 \end{array}\right]. \end{array}$$

**Step 3 .**

Calculate the distances of the positive and the negative ideal situation according to Definitions 1 and 2. Calculate positive and negative prospect value matrix taking the distances of the positive and the negative ideal situation as different reference points. The matrix for normalized ${\overset{-}{v}}^{\pm }[s_{ij}^{\pm 1(1)}]$ is as follows:

(13) $$\begin{array}{c} {\overset{-}{v}}^{+}\left[s_{ij}^{-1(1)}\right]=\left[\begin{array}{cccc} 0.6516 & 0.6999 & 0.5159 & 1\\ 0.3803 & 0.4926 & 0.4926 & 0.2541\\ 0.5159 & 0.4926 & 0.8482 & 0.6516 \end{array}\right],\\ {\overset{-}{v}}^{-}\left[s_{ij}^{-1(1)}\right]=0,\\ {\overset{-}{v}}^{-}\left[s_{ij}^{+1(1)}\right]=\left[\begin{array}{cccc} -0.6345 & -0.4926 & -0.6516 & -0.4677\\ -0.9495 & -0.7856 & -0.7856 & -1\\ -0.6516 & -0.7856 & -0.5159 & -0.6345 \end{array}\right],\\ {\overset{-}{v}}^{-}\left[s_{ij}^{+1(1)}\right]=0. \end{array}$$

Other normalized positive and negative prospect value matrixes can be gotten in the same way.

**Step 4 .**

The weight vectors of situation given by multiple experts are *W* ^1^ = [0.4,0.3,0.3], *W* ^2^ = [0.5,0.25,0.25], and *W* ^3^ = [0.5,0.2,0.3]. Plug those objective weight vectors into formula (5) and get comprehensive prospect value based on two reference points of the positive and the negative ideal situation distances. Consider

(14) $$\begin{array}{c} V_{ij}^{1}=\left[\begin{array}{cccc} 0.0598 & 0.3783 & -0.0239 & 0.2401\\ -0.6592 & -0.3916 & -0.3653 & -0.6721\\ -0.0976 & -0.4398 & 0.0679 & -0.1091 \end{array}\right],\\ V_{ij}^{2}=\left[\begin{array}{cccc} 0.0084 & 0.3999 & -0.1129 & 0.4052\\ -0.7221 & -0.3550 & -0.4722 & -0.5486\\ -0.1765 & -0.2935 & 0.2253 & -0.0399 \end{array}\right],\\ V_{ij}^{3}=\left[\begin{array}{cccc} -0.1744 & 0.3345 & 0.0537 & 0.3785\\ -0.7625 & 0.4141 & -0.5374 & -0.5472\\ 0.1359 & -0.3061 & 0.2095 & -0.1347 \end{array}\right]. \end{array}$$

**Step 5 .**

Determine the optimal and the worst prospect value matrix based on decision experts' judgment according to Definition 3 and calculate the distances between the prospect value matrix of multiple decision experts' situation and the optimal and the worst prospect value matrix according to Definition 4. Consider

(15) $$\begin{array}{c} d\left(V_{}^{1},V_{}^{+}\right)=0.7993,\;\;d\left(V_{}^{1},V_{}^{-}\right)=0.1119,\\ d\left(V_{}^{2},V_{}^{+}\right)=0.7351,\;\;d\left(V^{2},V_{}^{-}\right)=0.1426,\\ d\left(V_{}^{3},V_{}^{+}\right)=0.1093,\;\;d\left(V^{3},V_{}^{-}\right)=0.8472. \end{array}$$

**Step 6 .**

Calculate the weight of experts' strategy according to formulas (8) and (9) as *λ*_1_ = 0.4796, *λ*_2_ = 0.4579, *λ*_3_ = 0.0625. The comprehensive prospect value matrix of situation based on decision experts weight vector is as follows:

(16) $$\begin{array}{c} V=\left[\begin{array}{cccc} 0.0216 & 0.3855 & -0.0598 & 0.3243\\ -0.6945 & -0.3245 & -0.4250 & -0.6077\\ -0.1191 & -0.3645 & 0.1488 & -0.0790 \end{array}\right]. \end{array}$$

According to comprehensive prospect value matrix, choosing supplier 2 is the optimal strategy for production line 1; choosing supplier 2 is the optimal strategy for supplier 2; choosing supplier 3 is the optimal strategy for supplier 3; providing production line 1 is the most appropriate for supplier 1; providing production line 1 is the most appropriate for supplier 2; providing production line 3 is the most appropriate for supplier 3; and providing production line 1 is the most appropriate for supplier 4. In a word, the optimal situation is *S*_12_.

According to the methods used to calculate grey situation in group decision-making in [3], assuming that the experts' knowledge is the same (i.e., the expert's weight is equal), the weight vectors of the situation given by multiple experts are as follows: *W* ^1^ = [0.4,0.3,0.3], *W* ^2^ = [0.5,0.25,0.25], *W* ^3^ = [0.5,0.2,0.3].

The comprehensive effect sample value matrix of situation based on normative approaches given by [4] is as follows:

(17) R=[[0.179,0.269][0.235,0.342][0.187,0.293][0.221,0.330][0.183,0.304][0.204,0.348][0.197,0.346][0.189,0.324][0.193,0.299][0.169,0.289][0.149,0.379][0.189,0.301]].

According to the effect sample value matrix without the decision makers' behavioral preference, the optimal situation is *S*_12_ as well. However, the experts' weights are relying on the subjective experience and judgment. If the experts' weights are different, there may be different results.

From the example analysis and comparison, the method studies the group decision problem with a view to decision experts' risk attitude and effect sample value, considers decision experts' decision-making psychology, takes reference points as the positive and the negative ideal situation group distances, gets comprehensive prospect value matrix under multiple experts' evaluation, plugs the decision experts' risk preference into grey situation group decision, and makes sure that the final decision conforms to the psychology behavior of decision experts. Meanwhile, determine multiple decision experts' weight based on TOPSIS method by considering the close degree between expert's individual evaluation and overall assessment. Compared with the former subjective value methods, this method is more objective and is almost not relying on subjective experience and judgment.

## 4. Conclusion

This paper puts forward a grey situation group decision-making method based on the prospect theory, aiming at solving such problem that the grey situation group decision model does not consider the decision experts' risk preference. According to various types of effect sample value, different prospect value functions are constructed, the value functions for two reference points are integrated, strengths and weaknesses are measured by means of prospect value, and the prospect value is normalized to [−1,1]. This paper determines multiple decision experts' weight by means of TOPSIS method, collects experts' opinion, makes sequence for each event's situation, and gets the optimal situation at last. This method is convenient for computer procedure operation and provides a new method to solve problem of grey group situation taking decision experts' risk preference into consideration. However, the parameters of the prospect value functions and the weight functions are usually gained by experiments. The determination of the reasonable parameters still needs to be discussed and further researched. In view of the fact that decision experts discuss again and again evaluation process and modify assessment information, further study on multiround interactive grey situation group decision is needed.
